# Generation of Reactive Oxygen Species (ROS) by Harmful Algal Bloom (HAB)-Forming Phytoplankton and Their Potential Impact on Surrounding Living Organisms

**DOI:** 10.3390/antiox11020206

**Published:** 2022-01-22

**Authors:** Kichul Cho, Mikinori Ueno, Yan Liang, Daekyung Kim, Tatsuya Oda

**Affiliations:** 1 Department of Microbiology, National Marine Biodiversity Institute of Korea (MABIK), Seocheon 33662, Korea; kichul.cho@mabik.re.kr; 2 Graduate School of Fisheries Science & Environmental Studies, Nagasaki University, 1-14 Bunkyo-machi, Nagasaki 852-8521, Japan; mikiueno@nagasaki-u.ac.jp (M.U.); bb53419804@ms.nagasaki-u.ac.jp (Y.L.); 3 Daegu Center, Korea Basic Science Institute (KBSI), Daegu 41566, Korea

**Keywords:** reactive oxygen species (ROS), marine microalgae, harmful algae bloom (HAB) species, *Chattonella*, nitric oxide (NO)

## Abstract

Most marine phytoplankton with relatively high ROS generation rates are categorized as harmful algal bloom (HAB)-forming species, among which *Chattonella* genera is the highest ROS-producing phytoplankton. In this review, we examined marine microalgae with ROS-producing activities, with focus on *Chattonella* genera. Several studies suggest that *Chattonella* produces superoxide via the activities of an enzyme similar to NADPH oxidase located on glycocalyx, a cell surface structure, while hydrogen peroxide is generated inside the cell by different pathways. Additionally, hydroxyl radical has been detected in *Chattonella* cell suspension. By the physical stimulation, such as passing through between the gill lamellas of fish, the glycocalyx is easily discharged from the flagellate cells and attached on the gill surface, where ROS are continuously produced, which might cause gill tissue damage and fish death. Comparative studies using several strains of *Chattonella* showed that ROS production rate and ichthyotoxicity of *Chattonella* is well correlated. Furthermore, significant levels of ROS have been reported in other raphidophytes and dinoflagellates, such as *Cochlodinium polykrikoides* and *Karenia mikimotoi*. *Chattonella* is the most extensively studied phytoplankton in terms of ROS production and its biological functions. Therefore, this review examined the potential ecophysiological roles of extracellular ROS production by marine microalgae in aquatic environment.

## 1. Introduction

Over the years, hundreds of marine microalgae, which constitute a significant portion of marine biomass, have been described worldwide. Although several microalgae are known as promising sources of beneficial bioactive compounds, such as anti-cancer and antioxidant agents [1,2], less than 2% of them have been classified as harmful or toxic species [3]. The blooming of dominant algae species at relatively high cell density results in a phenomenon called “red tides”. In the case of harmful or toxic species, it is known as harmful algal blooms (HABs) [4]. The mass growth of specific algal species is easily recognized, often leading to water discoloration [4]. Most HAB species in marine environments are unicellular phytoplankton. HAB is a diverse phenomenon, involving multiple species and classes of microalgae that produce a wide variety of toxins or bioactive compounds, which can negatively affect several aquatic organisms [4,5,6]. Owing to advances in monitoring, surveillance, and identification technology, there has been an increase in the number of identified harmful and toxic microalgae species. Moreover, there has been an increased awareness of HABs due to an increase in the utilization of coastal areas for aquacultural activities [7,8]. Globalization of logistics seems to contribute to the distribution of HAB species to new areas through ballast water of ships or contaminated seafoods [9,10,11,12]. Recently, there has been an increase in the impact of HABs on fisheries and aquaculture industries. Moreover, the influence of HABs on marine ecosystems and organisms is projected to increase under the present climate change scenario, which could lead to considerable economic and ecological implications.

HABs have considerable implications on commercial and recreational fisheries and coastal tourism, as well as human and wildlife health [3,13,14,15,16,17].

There is emerging evidence that global warming-induced environmental changes may influence the patterns, frequency, distribution, and intensity of HABs in marine, brackish, and freshwater environments [18,19,20,21]. Changes in temperature [13], ocean acidification [22], nutrient conditions [23], and the physical structure of the water column [24], have been shown to influence the incidence and global range of HABs.

One of the significant impacts of HABs is the accumulation of algal toxins in shellfish, resulting in food poisoning of humans and animals. Symptoms of shellfish poison in humans include paralytic, diarrhetic, neurotoxic, amnesic, and azaspiracid poisoning [25]. Additionally, toxins and other bioactive compounds produced by algal species during bloom can cause the death of marine organisms. Furthermore, HABs can cause the death of wildlife, including seabirds, whales, dolphins, and other marine animals, through the transfer of toxins via the food web or direct ingestion of toxins [3,16]. Major incidences of HABs-associated mass mortalities and huge economic losses are summarized in Table 1. Although most of the toxins responsible for shellfish poisoning are well defined, the mechanisms of lethal action of harmful species are poorly understood. Overall, raphidophytes and dinoflagellates are the main groups of HAB species. Particularly, species of the *Chattonella* genera (*C. marina*, *C. antiqua*, *C. subsalsa*, *C. minima*, and *C. ovata*) have been reported to cause severe mortality of farmed organisms in mainly temperate and adjacent waters [26,27,28,29,30,31,32]. Yellowtail (*Seriola quinqueradiata*), atlantic salmon (*Salmo salar*), northern bluefin tuna (*Thunnus orientalis*), and bluefin tuna (*Thunnus maccoyi*i) are especially susceptible to *Chattonella* [32,33,34,35,36,37]. Mortalities have also been reported in benthic organisms, such as blue crabs, clams, octopus, pen shells, shrimps, and sea cucumber during *Chattonella* blooms [38]. In Japan, *Chattonella* have been reported to cause economic loss of approximately JPY 7.1 billion in the Harima-nada marine region in 1972 [39], and JPY 2.9 billion in 2009 and JPY 5.3 billion in 2010 in the Yatsushiro Sea [40]. Additionally, economic losses to fisheries due to *Chattonella* have been reported in other countries [25,30,31,32,35,37,38,41,42,43,44,45,46,47]. 

Although several studies have been performed on algal bloom, the exact toxic mechanisms of harmful algae are yet to be elucidated. Previous studies on the ichthyotoxic mechanism of *Chattonella* have identified several toxic or bioactive compounds involved in fish mortality [39,100,159,160,161,162,163,164,165] or the synergistic impact of multiple toxic factors [166]. It has been widely accepted that suffocation is the major cause of fish death by *Chattonella* [167,168,169,170]. Previous findings suggest that the direct target organ of *Chattonella* is the gill tissue, which can eventually lead to fish death. Matsusato and Kobayashi reported that the dead cells of *C. antiqua* and cell-free filtrate prepared from the live cell suspension of *Chattonella* was non-toxic to fish (red sea bream) [171]. Similarly, Ishimatsu et al. [172] also found that lysed cells of *C. marina* did not kill yellowtail. 

Other raphidophytes, such as *Heterosigma akashiwo*, *Olisthodiscus luteus*, and *Fibrocapsa japonica*, also produce extracellular ROS [85,88,160,173,174]. Thus, it seems that ROS production is a common biological feature of raphidophytes. However, extracellular ROS production is not limited to raphidophytes and has been reported in harmful dinoflagellates, including *Alexandrium* spp. [173,175,176,177], *Margalefidinium polykrikoides* (*Cochlodinium polykrikoides*) [62,178,179,180], and *Karenia mikimotoi* [176,181,182,183]. 

A recent review showed that phytoplankton are a major producer of ROS, including superoxide and hydrogen peroxide in aquatic environments [184]. Many phytoplankton taxa generate ROS under ordinal growth conditions without any stimuli or stress conditions. Although the physiological significance of extracellular ROS production by phytoplankton and their effects on the ecosystem remain unclear, the potential ecological and physiological effects of ROS production include biotoxicity, allelopathy, growth promotion, and iron acquisition. In this review, we described the levels, subcellular mechanism, biological roles, and toxic potential of ROS production by HABs species, with emphasis on the *Chattonella* genera [184]. Different assay methods have been applied for the detection of ROS produced by marine microalgae, as shown in Table 2.

Additionally, several lines of evidence suggest that *C. marina* can produce nitric oxide (NO) [185], which is involved in various important biological processes in mammals [186], several metabolisms of plants, expression of gene [187], and infectious diseases in plants [188,189]. Moreover, NO and superoxide can form peroxynitrite, a potent oxidant, by reacting to each other. Thus, the mechanism of NO production by *Chattonella* and other species and their biological activities was discussed in the later part of this review. 

## 2. Marine Microalgae Species with ROS-Producing Activities

In 1989, Shimada et al. [205] reported the first evidence of ROS production by the raphidophycean flagellate *Chattonella antiqua*. Since then, numerous studies have been conducted on *Chattonella* spp., including the role of ROS as an ichthyotoxic factor, ROS production mechanism, and the biological roles of ROS in *Chattonella* [28,31,206,207]. Despite extensive studies [86,88,93,100,159,160,166,171,208,209,210,211,212], the exact mechanism through which *Chattonella* cause fish death is still poorly understood. Recently, Shikata et al. [213] examined the ichthyotoxicity of eight strains of *Chattonella* with different backgrounds against different fish species (red sea bream and yellowtail) and found that the generation level of superoxide was most well-correlated with fish-killing activity among several factors examined, which supports the notion that ROS are mainly involved in the *Chattonella*-related fish mortality.

Moreover, few studies have found that other raphidophytes [85,88,173,174] and some dinoflagellates [62,173,175,176,177,178,179,180,181,182,183] are capable of producing ROS. Furthermore, Marshall et al. [173] examined the superoxide-producing ability of 37 species of microalgae, such as dinoflagellates, raphidophytes, and others, using chemiluminescence analysis, and found that several phytoplankton species are capable of producing superoxide to some extent. Detailed analyses showed a direct correlation between cell size and superoxide production level. Among the species, *Chattonella* produced the highest levels of superoxide per cell, whereas harmless species, such as *Dunaliella*, *Tetraselmis*, *Nannochloropsis*, and *Pavlova*, which are usually used as bivalve feeds, did not produce significant levels of ROS. Furthermore, based on the degree of superoxide production and toxicity, they proposed that phytoplankton species could be classified into four groups. Microalgae producing ROS with exceled certain threshold value, such as *C. antiqua*, *C. marina*, *C. minima* and *C. ovata*, were categorized as extremely toxic. 

According to a review paper on the diversity of phytoplankton in aquatic environments [184], the generation rate of ROS per cell was measured in more than 21 microalgae species; most of them were HAB-forming species [62,85,173,175,182]. The generation rate of one ROS (superoxide; O_2_^−^) has also been quantified in some species of cyanobacteria [148,199,214,215,216]. Furthermore, HAB species produce higher levels of O_2_^−^ than other phytoplankton taxa, including freshwater cyanobacterium *Microcystis aeruginosa* [216], and non-harmful species [196]. These findings indicate that various phytoplankton species are major biological sources of ROS in marine environment, which can cause profound ecological impact on marine environment. Considering the high level of ROS production in marine environments, it is necessary to examine the effects of ROS produced by HAB species, including raphidophytes and dinoflagellates, on marine organisms. Therefore, *Chattonella* spp., other raphidophytes, and dinoflagellates, such as *Cochlodinium polykrikoides* and *Karenia mikimotoi*, were discussed comprehensively in subsequent sections. Table 3 shows details of high ROS-producing marine phytoplankton species.

## 3. Chattonella

Raphidophycean flagellates *Chattonella* spp (*C. marina*, *C. antiqua*, *C. subsalsa*, *C. minima*, and *C. ovata*) are causative species of HAB-associated fish mortality, with serious impact on the aquacultural industry in Japan [31]. Among the genus, *C. marina* and *C. antiqua* are highly toxic species, which are causing enormous negative impact on fish farms in Japan, particularly to yellowtail (*Seriola quinqueradiata*) aquaculture in the last few decades [212]. Additionally, fish mortality due to *Chattonella* spp-induced HABs has occurred in Australia, Netherlands, Brazil, and other parts of the world [28,31,206,207].

Previous studies have proposed several potential toxic factors, such as neurotoxins resembling the brevetoxins produced by *Karenia brevis* (formerly known as *Gymnodinium breve* and *Ptychodiscus brevis*), haemagglutinating agents [86,93,100,210,211], fatty acids [166,212], and mucus substances [171]. Moreover, Shimada et al. [163] reported that *C. antiqua* has the ability to induce SOD-inhibitable cytochrome c reduction, indicating that live *C. antiqua* cells can produce superoxide anion. Further studies using several techniques demonstrated that *Chattonella* spp. generate ROS, such as superoxide (O_2_^−^), hydrogen peroxide (H_2_O_2_), and hydroxyl radical (•OH) [88,159,160,208,209]. Since ROS are biologically highly toxic [224,225], *Chattonella* spp. may exert ichthyotoxicity through ROS at least in part. This hypothesis might be supported by the low toxicity of low superoxide-producing *C. marina* strains [172,226]. Similarly, Cho et al. [164] reported relatively high ichthyotoxicity of high ROS-producing *C. antiqua* strains compared with low ROS strain of *C. marina*. In addition to *Chattonella* spp., another raphidophycean flagellate, *Heterosigma akashiwo*, has been reported to show ROS-mediated toxicity against rainbow trout [85]. It is widely accepted that suffocation is the main mechanism of fish death by these flagellates [167,168,169,170], with loss of branchial respiratory capacity as an immediate physiological change observed in fish after exposure to *Chattonella* spp. [31,227]. Exposure of *S*. *quinqueradiata* to *C. marina* at a lethal cell density causes a rapid decrease in the arterial oxygen pressure within less than 30 min [168,169,228], resulting in further physiological responses, such as acidosis [229], ionoregulatory failure [168], increase in circulating catecholamine levels [230], and decrease in cardiac output [228]. Owing to a decrease in arterial oxygen pressure, excess mucus-like substances are secreted, probably by gill tissues, in response to stimulus by *C. marina*, which cover the gills. Such mucus substance on the gill surface together with glycocalyx, a polysaccharide-containing complex cell surface structure discharged from the flagellate cells, may interfere with O_2_ uptake from gill lamellas, resulting in asphyxia [169,231,232]. A further in vitro study demonstrated that there was a 26–83% decrease in water flow rate through the excised first gill arch of jack mackerel (*Trachurus japonicus*) placed in 4000 cells of *C. marina*/mL for 10 min compared with those placed in culture medium alone, and the gill arch of the flagellate-exposed group was covered with mucus and *C. marina* cells [233]. In mammalian systems, ROS enhance mucus secretion from various epithelia lining luminal organs, such as the gallbladder of guinea pigs in vitro [234,235], the gastric mucous cells of rats [236], and the tracheal epithelial cells of guinea pigs [237,238]. Considering the similarities between fish and mammals in terms of mucins, mucus cells [239], and secretory mechanisms [240], it could be speculated that ROS produced by *C. marina* may be involved in the over secretion of mucus on the gill tissue. 

Based on previous findings, it seems likely that live cell condition is important for the ichthyotoxicity of *Chattonella*. Matsusato and Kobayashi [171] reported that neither the dead cells of *C. antiqua* nor cell-free supernatant of the flagellate culture were toxic to fish. Similarly, Ishimatsu et al. [172] reported that ruptured *C. marina* showed no toxic effect on yellowtail and found that there is a clear correlation between the cellular O_2_^−^producing activity and its fish toxicity. The toxin may probably be quite unstable in nature, leading to the disappearance of its activity in ruptured cells and rendering the isolation of the toxin in its active form difficult. Considering these findings, among the toxic factors proposed, ROS is the most provable candidate.

Previous studies demonstrated that *C. marina* suppressed the growth of *Vibrio alginolyticus* inoculated into plankton culture [160]. The bactericidal activity of *C. marina* was significantly suppressed by superoxide dismutase (SOD) and catalase, which are antioxidant enzymes with ROS scavenging activity. Additionally, sodium benzoate, a hydroxyl radical scavenger, protected the bacteria from the toxic effect of *C. marina*. Thus, it was suggested that *C. marina* can exert negative impact on surrounding bacteria through ROS production. This is an indicative example that ROS-producing marine microalgae, such as *Chattonella* spp., can cause oxidative stress to surrounding organisms.

### 3.1. Mechanisms of ROS Production by Chattonella

Various phytoplankton species produce extracellular ROS under normal growth conditions, with *Chattonella marina* being the highest extracellular ROS producer [184]. ROS production by *Chattonella* has been well documented in several independent studies [159,160,161,163,164,226]. Several techniques have been employed for the detection of each reactive oxygen species; superoxide anions (O_2_^−^) by cytochrome c reduction [160], chemiluminescence analysis [164], and fluorescent microscopy [162,163]; hydrogen peroxide (H_2_O_2_) by the phenol red or the scopoletin assay [161]; and superoxide and hydroxyl radicals (•OH) by electron spin resonance spectroscopy [159,164]. In *Chattonella* cells, ROS production can occur in several major organelles or intracellular compartments, such as chloroplasts, mitochondria, peroxisomes, and cell membrane. The primary oxygen radical-producing step at these sites is the formation of O_2_^−^ via the single electron reduction of O_2_, and the subsequent enzymatic or non-enzymatic dismutation of superoxide is the most probable mechanism for the production of H_2_O_2_. Intracellularly generated H_2_O_2_ might easily release extracellularly [94,162]. In contrast, O_2_^−^ is membrane impermeable in nature due to its short life span and limited diffusion distance, indicating that O_2_^−^ hardly crosses cell membranes [241,242]. Thus, the most probable site of O_2_^−^ generation in *Chattonella* may be on the cell surface. To identify the mechanism of O_2_^−^ and H_2_O_2_ production, especially focusing on intracellular location of O_2_^−^ and H_2_O_2_ production in *C. marina* and *C. ovata*, Kim et al. [162] conducted fluorescence microscopic observation of these flagellate cells using methyl cypridina luciferin analog and 5-(and-6)-carboxy-20,70-dichlorodihydrodihydrofluorescein dictate, acetyl ester, which is a specific fluorescent probe for detecting O_2_^−^ and H_2_O_2_, respectively. The fluorescence pictures suggested that superoxide is produced on the cell surface, whereas hydrogen peroxide is produced intracellularly. Furthermore, destruction of the cells by ultrasonic treatment resulted in significant decrease in O_2_^−^ levels, whereas the level of H_2_O_2_ detected in the ruptured cells increased as compared to the level of intact cell suspension [161,162]. Thus, the producing mechanisms of O_2_^−^ and H_2_O_2_ and their intracellular location seem to be different and independent of each other in the cells. 

Generally, it has been considered that hydroxyl radical is the most toxic radical that can destroy proteins, nucleic acids, and other important biomolecules [224,243]. Since hydroxyl radical is detected in the flagellate cells, the ecological impact of these ROS-producing flagellates should be significant. The reaction of superoxide radical and hydrogen peroxide can produce hydroxyl radical. For this reaction, transition metals, such as Fe^2+^ and Cu^2+^, play an important role as reducing agents in the Fenton reaction and the Haber–Weiss cycle [224,243]. Iron is generally required for optimal growth of phytoplankton [244], and the flagellate culture medium contains 0.5 μM EDTA-Fe^3+^ and certain levels of other metal ions. Thus, it is possible that the hydroxyl radical is produced through the Fe-catalyzed Fenton-type Haber–Weiss reaction. This assumption was supported by the fact that hydroxyl radical production in flagellate cell suspension is inhibited by either SOD or catalase [159]. To further evaluate the roles of iron or other metals in hydroxyl radical production, the effect of hypoxanthine/xanthine oxidase addition as a superoxide generation system to flagellate culture medium was examined using electron spin resonance (ESR) analysis, and the results showed that hypoxanthine/xanthine oxidase remarkably increased hydroxyl radical production [159]. Because Fe exists in seawater in the 0.01–1 μM range [245], the formation of hydroxyl radical by *Chattonella* spp. in seawater is feasible.

Tang et al. [246] showed that H_2_O_2_ alone was not lethal to fish. It should be noted that *Chattonella* cells produce both superoxide anions and hydrogen peroxide, and the co-occurrence enhances *Chattonella* toxicity to living organisms, which could be due to hydroxyl radical formation [247,248]. Additionally, studies have shown that H_2_O_2_ had no effect on mucin release in several cell culture or explant models [235,237,249], whereas hydroxyl radical induced mucus secretion [235]. Since the presence of excessive mucus substance on the gill surface of fish exposed to *Chattonella* is considered as a key factor, hydroxyl radical may play a major role among the ROS in terms of ROS-mediated detrimental effect of *Chattonella* on fish gill.

### 3.2. NADPH Oxidase as a Superoxide-Anion-Producing Enzyme System

In various biological systems, the generation of extracellular superoxide anion (O_2_^−^) is likely regulated by enzyme systems, such as oxidoreductases, utilizing nicotinamide adenine dinucleotide phosphate (NADPH), which acts as a reducing co-factor for the conversion of O_2_ to O_2_^−^. An enzyme NADPH oxidase existing in the plasma membrane of certain white blood cells catalyzes the single-electron reduction of O_2_ to O_2_^−^ [250]. In higher plant cells, there are some NAD(P)H oxidase capable of generating O_2_^−^ in the plasma membranes, and ROS production in plant cells shows similar characteristic of ROS-generation system so-called oxidative burst in mammalian phagocytic cells [251]. 

Raphidophytes (*C. antiqua*, *C. marina*, *H. akashiwo*) possess glycocalyx as a cell surface structure [232,252,253,254], and enzymatic system responsible for O_2_^−^ generation exists in the glycocalyx, which is easily dissociated from the cells under physical or chemical stimulation [97,191]. To determine the involvement of glycocalyx in ROS generation in *C. marina*, Kim et al. obtained a supernatant from a *C. marina* cell suspension by mild agitation, which caused discharge of the glycocalyx without cell destruction [94]. Chemiluminescence assay using O_2_^−^ specific probe showed that the cell-free supernatant induced SOD-inhibitable strong chemiluminescence in response to exogenous NADPH, whereas the supernatant without NADPH showed only a trace-level response. Additionally, concentration of high molecular weight fraction by ultrafiltration resulted in increased chemiluminescence response, suggesting that certain components with large molecular size in the supernatant are responsible for the reaction. On the other hand, *C. marina* cell suspension exhibited no response to NADPH. Since NADPH is not membrane permeable, *C. marina* might not be able to utilize extracellular NADPH. NADH was less effective as compared to NADPH, and NADP^+^ and NAD^+^ were ineffective. In addition, diphenyleneiodonium, an inhibitor of mammalian NADPH oxidase, prevented NADPH-induced chemiluminescence response in the cell-free supernatant. Probably, *C. marina* has an enzyme system similar to NADPH oxidase of neutrophil. NADPH oxidase of neutrophil has two subunit proteins in the plasma membrane, gp91phox and p22phox, which form heterodimeric flavocytochrome b558 [250]. To further clarify the O_2_^−^-generating enzyme system in *C. marina* cells, immunoblotting of the cell-free supernatant of *C. marina* was performed using an antibody raised against neutrophil gp91phox. The result suggested the presence of protein recognized with the antibody in the cell-free supernatant of *C. marina*. Additionally, indirect immunofluorescence of the flagellate cells using the same antibody indicated that human gp91phox-like protein existed on the surface of *C. marina*. Furthermore, southern blot using the oligonucleotide probe encoding the C-terminal region of human gp91phox suggested the presence of a gene encoding a protein mimicking gp91phox in *C. marina*. In addition to several well-described mammalian homologs of gp91phox [255,256,257], higher plant cells (*Arabidopsis thaliana*) have slightly larger homologs of gp91phox with 59.8–62.3% sequence similarity to gp91phox [258]. 

The presence of NADPH oxidase in *Chattonella* as a source of O_2_^−^ is further supported by the identification of six putative genes encoding NADPH oxidase (NOX) in *C. antiqua* [259]. The enzymatic activity of NOX requires NADPH, which is mainly supplied by the oxidative pentose phosphate (OPP) pathway [260,261]. Regarding the regulation mechanism of NOX activity in *Chattonella*, it has been observed that the production of O_2_^−^ in *C. marina* and *C. antiqua* is inhibited by an inhibitor of photosynthetic electron transport, 3-(3,4-dichlorophenyl)-1,1-dimethylurea [193,262]. These findings suggest that both photosynthesis and OPP pathways are involved in the production of O_2_^−^ in these flagellate cells. Interestingly, a recent study found that O_2_^−^ production in *C. antiqua* increased under nutrient deficiency and suppressed photosynthesis conditions, suggesting that increases in the ratio of NADPH to NADP^+^ caused by the OPP pathway might be deeply involved in ROS generation in *Chattonella* [263].

### 3.3. Glycocalyx as a Cell Surface Structure with ROS Generation System

Electron and light microscopic observation of *Chattonella antiqua* and *Heterosigma akashiwo* showed that these cells have glycocalyx as the cell surface, which consists of sulfated, non-sulfated polysaccharides, and neutral carbohydrate–protein complex [252,253]. Since raphidophycean flagellates generally do not have a rigid cell wall, glycocalyx may function as a defense or barrier against biological and non-biological invasion. Oda et al. [191] showed that the addition of lectins, such as concanavalin A (Con A), wheat germ agglutinin, and castor bean haemagglutinin, significantly increased in O_2_^−^ generation by *C. marina* and *H. akashiwo*. Since the effects of the lectins were suppressed by specific monosaccharides, the binding of the lectins to the saccharide moieties on the cell surface may have led to increased O_2_^−^ production. Interestingly, high concentration of Con A can induce morphological changes in these flagellate cells. After the addition of Con A, some cells became spherical, distinct from the usual spindle shape, and these changes were frequently accompanied by the discharge of glycocalyx. An analysis using fluorescent-labeled Con A confirmed the binding of Con A to the discharged glycocalyx. These results suggest that the binding of Con A to the glycocalyx is recognized as a stimulus by the flagellate cells, leading to discharge of glycocalyx. Shimada et al. [163] and Tanaka et al. [99] reported that O_2_^−^ was generated in small particles, or in verruciform protrusions located on the cell surface of *C. antiqua*. Additionally, the addition of mucus substances prepared from yellowtail induced the release of these small particles from the flagellate cells. Similarly, Nakamura et al. [218] and Okamoto et al. [254] observed that extracellular addition of mucus substances obtained from yellowtail gill enhanced O_2_^−^ generation by *C. marina*, which was concomitant with the discharge of the glycocalyx. The presence of O_2_^−^ generation system on the glycocalyx, may be supported by the observed suppression of O_2_^−^ generation by *C. marina* and *H. akashiwo* treated with membrane-impermeable protease [191]. In fish mucus, lysozyme, proteases, and lectins and other bioactive molecules have been discovered, as well as mucin, a major mucus comportment [264,265,266]. Some components in fish mucus that possess lectin activity may act as a stimulus mimicking Con A and induce glycocalyx discharge and activate O_2_^−^ generation. 

In addition to lectins or mucus, simple agitation seems to influence the glycocalyx. Matsusato and Kobayashi [171] argued that *Chattonella*-mediated fish mortality could be due to inhibition of respiratory water flow through the gills by mucus substance derived from the flagellate cells. It has been reported that *Chattonella* can secret mucus substances when the cells were passed through a net with 95 μm mesh size. Thus, it could be inferred that mucous substances on the gill surface of fish exposed to *Chattonella* are at least partly derived from *Chattonella* cells. Moreover, indirect immunofluorescence using antiserum raised against crude glycocalyx of *C. marina* suggested the presence of glycocalyx, together with *C. marina* cells on the gill surface of fish exposed to *C. marina* [232]. 

Ishimatsu et al. [168,229] demonstrated that the earliest physiological and histological changes observed in the yellowtail after *Chattonella* exposure was a rapid drop of arterial oxygen partial pressure and considerable accumulation of mucous substances between the filaments and lamellae of the gill tissues, respectively. Based on the previous studies, it seems obvious that glycocalyx plays a pivotal role in the ichthyotoxic mechanism of *Chattonella* and other raphidophycean flagellates. 

## 4. Raphidophycean Flagellates

*Heterosigma akashiwo*, *Olisthodiscus luteus*, and *Fibrocapsa japonica* often cause serious mortality of wild and farmed fish [267]. Generation rates of superoxide anion (O_2_^−^) and hydrogen peroxide (H_2_O_2_) by *C. marina* (two strains), *C. antiqua*, *H. akashiwo*, *O. luteus*, and *F. japonica* were estimated by SOD-inhibitable cytochrome c reduction and scopoletin assay, respectively [88]. *Chattonella* showed the highest O_2_^−^ and H_2_O_2_ production rates among the raphidophytes tested, based on cell number. This may be due to different cell sizes. *Chattonella* has nearly ten times larger cell size than other raphidophycean flagellates.

Interestingly, an increase in H_2_O_2_ levels of disrupted cell suspensions of these raphidophytes even higher than intact flagellate cell suspensions was observed [161]. These findings suggest that these raphidophytes have a certain intracellular compartment where H_2_O_2_ might be accumulated at high concentration, from which it is gradually released into the medium during normal growth. The presence of intracellular compartment with high H_2_O_2_ concentration may be a common cellular feature of raphidophytes. Regarding the behavior of H_2_O_2_, it has been demonstrated that there was a decrease in the concentration of exogenously added H_2_O_2_ in *C. marina* cell suspension, with approximately 30 min half-life, whereas H_2_O_2_ in the culture medium alone was much more stable [161].

## 5. Cochlodinium Polykrikoides

*Cochlodinium polykrikoides* is a harmful dinoflagellate with potent fish-killing activity [59,90,268]. Apart from *C. polykrikoides*, *C. fulvescens* [59,269,270] and *Cochlodinium* sp. Type Kasasa [271] have been identified as morphologically similar and toxic species. Cells with 28–35 μm diameter form 4–8 chains, depending on growth conditions. *Cochlodinium* blooms have been reported in Japan, Korea, and other countries [272,273], and cause fish mortality [269,274]. For instance, *C. polykrikoides* bloom caused economic losses of more than USD 100 million to fisheries in Korea [62,275].

Previous studies reported that certain toxic compounds, including neurotoxin, hemolytic toxin, haemagglutinative agent, and paralytic shellfish poisoning (PSP) toxins were found in *C. polykrikoides* [3,58,61,276]. Kim et al. reported that *C. polykrikoides* isolated in Korea generated O_2_^−^ and H_2_O_2_ [62] and proposed that *C. polykrikoides* exerts gill tissue damage and fish mortality through ROS production [277]. Contrarily, *C. polykrikoides* isolated in Japan produced O_2_^−^ and H_2_O_2_ with much lower levels than those of *Chattonella marina*, and the strains isolated in Japan did not respond to lectins and fish mucus [178]. Further studies showed that only trace levels of ROS were detected in cell suspensions of five clonal strains of *C. polykrikoides* isolated from different localities, and at times in Japan. To evaluate the fish-killing activity of the Japanese strains of *C. polykrikoides* with low ROS generation activity, damselfish (*Chromis caerulea*; average length 3 ± 0.8 cm) were exposed to the flagellates (4 × 10^3^ cells/mL), and the results showed that damselfish were susceptible to all the strains of *C. polykrikoides* tested, with 100% mortality within 90 min of exposure, whereas no significant protective effects of SOD and catalase were observed in the exposure experiments [194]. The reason for the discrepancy between the Korean and the Japanese strain of *C. polykrikoides* is still unclear. However, since ROS level is dependent on strain, growth conditions, and assay methods [88,173,184], it is possible that the Korean strain may have an extremely more potent ROS generation activity than the Japanese strains. In addition, there is a possibility that certain toxic factors other than ROS might be mainly involved in the ichthyotoxicity of *C. polykrikoides*. Regarding the fish-killing mechanism of *C. polykrikoides*, suffocation caused by huge amount of mucus substances derived from *C. polykrikoides* could be responsible for fish death [61,276]. Exposure experiments using several fish species demonstrated that still-unknown toxic agents together with mucus substances secreted from *C. polykrikoides* may be responsible for fish mortality [269]. Furthermore, *C. polykrikoides* continuously secretes large amounts of mucous substances into the medium as a characteristic feature [178]. Therefore, it could be inferred that the mucus and certain toxins, including ROS, may be involved in the fish-killing mechanism of *C. polykrikoides*. Interestingly, Shin et al. have reported that *C. polykrikoides* induced oxidative damage and DNA degradation in the gill of red seabream after exposure to sub-lethal concentrations of the flagellate [278]. 

## 6. Karenia Mikimotoi

The dinoflagellate *Karenia mikimotoi* (*K. mikimotoi*), formerly *Gyrodinium aureolum*, *G.* cf. *aureolum*, *G.* type-’65, *G. nagasakiense*, and *G. mikimotoi*, is highly toxic to both fish and shellfish [279]. *K. mikimotoi* is an unarmored dinoflagellate with average cell size of 23–40 μm in diameter and flattened, with a characteristic swimming [280]. *K. mikimotoi* is a eurythermal and euryhaline organism, which can survive at temperature range of 4–31 °C and salinity at 9–31 [281,282]. Additionally, *K. mikimotoi* grows under light intensities (10 to 1200 μmol/m^2^/s) and can assimilate different chemical forms of nitrogen and phosphorous [53,283]. 

HABs of *K. mikimotoi* have occurred in Japanese waters [15,284], the North Atlantic [53,285], and other areas [286,287]. HABs of *K. mikimotoi* have caused massive mortality of fish [15] and shellfish in Japan [288]. Since the mid-1960s, when *K. mikimotoi* bloom occurred in Japan [289], mortality of various fish and invertebrate species caused by *K. mikimotoi* has been reported in Europe, Australia, Japan, South America, and North Africa [3]. 

Regarding the toxic mechanisms of *K. mikimotoi*, it has been reported that *K. mikimotoi* produce several toxic agents, such as low-molecular-weight hemolytic toxins [290,291,292,293], cytotoxic polyethers [294,295], and ROS [181,296]. Matsuyama reported that *G. mikimotoi* strongly inhibited the filtration rate of bivalves [297]. Sellem et al. demonstrated that the 18:5n3 fatty acid produced by *G. mikimotoi* exhibited detrimental effects on sea urchin (*Paracentrotus lividus*) [298]. Mitchell and Rodger reported that *K. mikimotoi* bloom was associated with fish and shellfish mortality [299]. 

In 2012, large-scale HAB of *K. mikimotoi* (2 × 10^3^–1.18 × 10^5^ cells/mL) caused mass mortality of Japanese pufferfish (*Takifugu rubripes*) in Japan [300]. Exposure studies confirmed that NGU04, a strain of *K. mikimotoi* isolated from the HAB area, was toxic to fish during the time (0.3–4 h) in cell-density-dependent manner (5 × 10^2^–1 × 10^4^ cells/mL). Interestingly, NGU04 produced extremely high levels of ROS, which were nearly equal to the levels of *C. marina* measured at the same time [183]. ROS generation by *C. marina* increases in response to extracellular stimuli, such as lectins [191,218]. After binding to the cell surface carbohydrate moieties, lectins induce various cellular signaling pathways, leading to the enhancement of ROS generation in leukocytes [301,302]. *C. marina* might possess such pathways regulating ROS generation in response to lectin stimuli, as found in leukocytes. NGU04 also increased ROS levels in the presence of three lectins, which had different saccharide specificity. Additionally, the lectin response profile of NGU04 differed from that of *C. marina*. This may reflect differences in cell surface structures between raphidophycean flagellate *C. marina* and dinoflagellate *K. marina*, especially the lectin binding sites [183]. Regarding O_2_^−^ and H_2_O_2_ generation mechanisms, *Hymenomonas carterae*, a marine phytoplankton, produced extracellular H_2_O_2_ without utilizing O_2_^−^ [222]. Similarly, fluorescence microscopic observation of the NGU04, using ROS-specific fluorescence probes, indicated that O_2_^−^ and H_2_O_2_ are produced in different intracellular compartments [183].

The zooplankton *Brachionus plicatilis* (*B*. *plicatilis*) is highly susceptible to *K. mikimotoi* [303], with NGU04 exhibiting the most lethal effect against *B*. *plicatilis* among the *K. mikimotoi* strains tested, whereas *C. marina* had no significant effect on the zooplankton under the same experimental conditions [183]. Since the toxic potential of *K. mikimotoi* on marine organisms, including shellfish, is well correlated with its toxicity against rotifer [303,304], the response of rotifer to the algae could be a reliable assay for evaluating *K. mikimotoi* toxicity. Moreover, a recent review [305] indicated that rotifer could be useful for marine ecotoxicology studies. Thus, the results suggest that NGU04 can exert potent toxicity on shellfish, as well as on fish. As supporting evidence for this, NGU04 demonstrated that it was lethal against juvenile abalone (*Nordotis gigantea*) in laboratory experiments [306]. Furthermore, NGU04 exerted hemolytic activities against rabbit and fish erythrocytes, and the activities were much stronger than other strains of *K. mikimotoi* with different backgrounds [306]. Since antioxidant enzymes, such as SOD and catalase, had no effect on rotifer toxicity of NGU04, it could be inferred that ROS might not be the major toxic factor of the strain, at least against rotifer [183]. Therefore, it is possible that *K. mikimotoi* exerts its toxic effect against rotifer and shellfish mainly through its hemolytic activity. This notion is supported by the non-toxic effect of *C. marina*, with no hemolytic activity against rotifer; however, *Heterocapsa circularisquama* with potent hemolytic activity can kill rotifer and shellfish, but not fish. 

Overall, it could be concluded that *K. mikimotoi*, especially at high cell density blooms, can negatively affect several surrounding organisms, not only through ROS-mediated oxidative stress, but via its hemolytic activity.

## 7. Nitric Oxide (NO) Production in Marine Microalgae

Over the years, studies have shown that *C. marina* generates nitric oxide (NO) under ordinal growth conditions [185]. As a first experiment, chemiluminescence (CL) reaction between NO and luminol–H_2_O_2_ was employed to detect NO in *C. marina* [307]. When H_2_O_2_ and luminol were added to *C. marina* simultaneously, increased CL response was detected, and it was significantly suppressed by 2-(4-carboxyphenyl)-4,4,5,5-tetramethylimidazoline-1-oxyl-3-oxide (carboxy-PTIO), a specific NO scavenger [308]. Detailed kinetic analyses indicated that NO production by *C. marina* was cell-density-dependent, and the level of NO for 10^4^ cells/mL was estimated to be nearly 10 μM. Griess reaction also confirmed the NO production by *C. marina* [309]. Furthermore, NO was detected in *C. marina* cell suspension using NO-reactive fluorescent probe diaminofluorescein-FM diacetate (DAF-FM DA) technique; however, NO fluorescence was completely inhibited by carboxy-PTIO [310]. Moreover, fluorescence microscopic observation suggested that NO was generated intracellularly in *C. marina*, and bright fluorescence of *C. marina* was inhibited by carboxy-PTIO. In addition, a comparative study showed that significantly higher fluorescence was detected in raphidophytes *Chattonella ovata* and *Heterosigma akashiwo*, whereas only trace levels of fluorescence were observed in dinophytes *Alexandrium tamarense*, *A. taylori*, *Cochlodinium polykrikoides*, *Gymnodinium impudicum*, and eustigmatophycean *Nannochloropsis oculata*. These results suggest that relatively high level of NO production may be a common specific feature of the raphidophycean flagellates [204]. To analyze the mechanism of NO production in *C. marina*, the effect of an inhibitor of NO synthase (NOS), NG-Nitro-L-arginine methyl ester (L-NAME) was examined. L-NAME is known to block NO production in mouse macrophage cell line RAW264.7 cells [311]. Similarly, L-NAME inhibited NO production by *C. marina*. In contrast, the addition of L-arginine, a substrate for NOS, resulted in an increase in NO level, with results obtained by luminol–H_2_O_2_ assay. 

NO, a gaseous free radical initially described as an endothelium-derived relaxing factor [312], is involved in various biological processes in mammals [186]. NO plays numerous roles not only in animals but also in plants. NO is membrane permeable and is involved in many important processes in plants [187,188,189]. Apart from higher plants, green algae and cyanobacteria also produce NO [313,314,315]. Similarly to *C. marina*, some species of marine microalgae also generate NO under certain conditions [102,316,317]. The results obtained by three independent assay methods demonstrated that *C. marina* is capable of producing NO without specific stimuli or stress conditions. In mammalian systems, NO is mainly produced from L-arginine by NOS, which yields L-citrulline and NO [318]. In plants, nitrate reductase (NR) has been demonstrated as a major NO generation system [319]. Moreover, NO is also produced by non-enzymatic nitrite reduction at acidic conditions [320]. In contrast to *C. marina*, it has been reported that unicellular alga *Chlamydomonas reinhardtii* produces NO through NR activity [315], and not through the activity of NOS-like enzyme. As observed in *C. reinhardtii*, NO production by *Chlorella sorokiniana* occurred in darkness and required nitrite [321]. Furthermore, it has been suggested that the NR-mediated NO production activity of these microalgae was linked with photosynthetic electron transport system because illumination of the algae cells suppresses NO production; however, the suppressive effect can be reversed by 3,4-dichlorophenyl-1,1-dimethylurea, a photosynthesis inhibitor [315]. Thus, it is likely that these unicellular algae may have a common NO production mechanism that can be affected by various growth conditions. Regarding *C. marina*, continuous NO production was observed under normal growth conditions (under illumination), and exogenous nitrite had no effect, suggesting that NR may not be involved in NO generation. Similarly to *C. marina*, some evidence suggests the presence of NOS-like activities in photosynthetic organisms [316,322]. Furthermore, a pathogen-induced NO-synthesizing enzyme has been purified from tobacco leaves [323]. Such pathogen-induced NO production in plants may play an important role in defense mechanism against pathogens through hypersensitive response [189]. 

NO has been shown to be an important regulator of mucus secretion in the stomach [324,325,326], and NO donors induced mucus secretion from isolated gastric mucus cells [325]. Therefore, it is conceivable that NO alone or in combination with ROS may induce over-secretion of mucus substances on the gill surface of fish exposed to *C. marina* cells, leading to the blockage of respiratory water flow. Shimada et al. [327] observed the presence of highly concentrated NOx in the cortex of *Chattonella antiqua*, and they proposed that NOx may induce mucous discharge from gill surface when *C. antiqua* cells pass between gill lamellae. NO [328] and its oxidized stable product, nitrite [329], are also known to oxidize hemoglobin to methemoglobin that cannot transport oxygen, leading to tissue hypoxia [330]. Regarding histological findings in the gills of fish exposed to *C. marina*, it has been reported that *C. marina* induced mucus secretion and altered gill lamella integrity in goldlined seabream after exposure, but no significant increase in methemoglobin was observed in the fish, even after developing symptoms [331]. 

Apart from the possible factor involved in fish-killing mechanism of *C. marina*, NO concentration of marine environments is 10^4^ times higher than atmospheric NO level due to extensive photolysis of nitrate and nitrification processes [332], and some species of microalgae release NO during the natural growth process [102,317]. Overall, these findings suggest that NO could be a stressor of marine organisms in multiple ways. 

For a quick overview of this review, the most harmful and notable HAB-forming species, together with some topics specific to the species, are summarized in Table 4. *C. marina*, *C. antiqua*, *C. polykrikoides*, and *Karenia mikimotoi* are well-recognized ROS-producing harmful algae, and ROS seem to play pivotal roles in the ichthyotoxic mechanisms, while hemolysin might be responsible for shellfish toxicity. 

## 8. Conclusions

HABs are a serious threat to marine resources and fisheries. Anthropogenic changes, including global warming, can further increase the distribution of HABs and the appearance of new HAB species. However, studies are yet to comprehensively elucidate the toxic mechanism of HAB species. In this review, we focused on raphidophytes (*Chattonella marina*, *C. antiqua*, and *Heterosigma akashiwo*) and dinoflagellates (*Karenia mikimotoi* and *Cochlodinium polykrikoides*), which are the major groups of HAB species. Since HABs of these species frequently cause mortality of wild and farmed marine organisms, with huge economic losses (Table 4), it is necessary to have a comprehensive understanding of the HAB species, their toxic mechanisms, and their blooming period or conditions, which may help in minimizing their impact.

**Figure 1:**
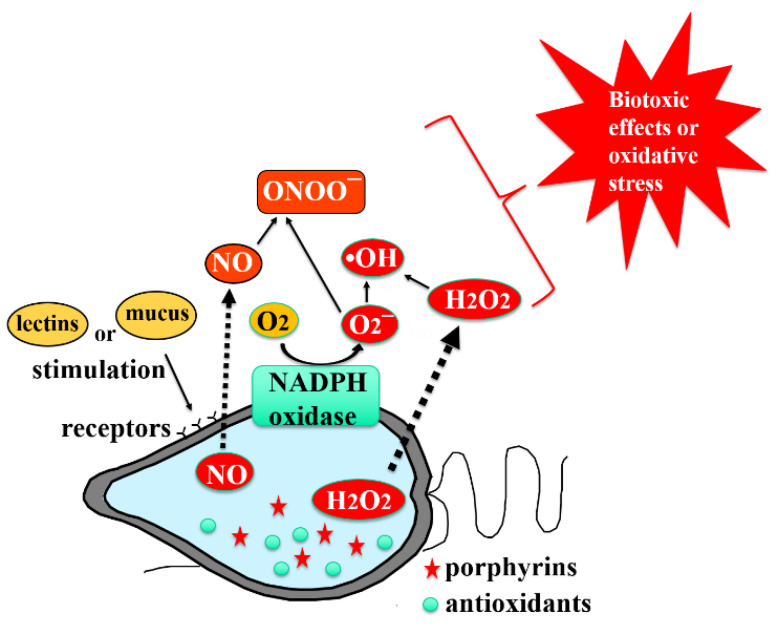
Production of reactive oxygen species (ROS) and other bioactive molecules in *Chattonella*.

The findings of this review showed that most of the harmful algae, especially the ichthyotoxic species described above, produced relatively higher levels of ROS, with *Chattonella* having the highest production rate. Although extensive studies are required to fully understand the biological significances of ROS production by HAB species and their impact on surrounding ecosystems and organisms, it is probable that ROS play pivotal roles in the fish-killing activities of HAB species, such as *Chattonella*, which is supported by the findings of previous studies. ROS production alone may not sufficiently explain the ichthyotoxic mechanism of the HAB species and could be attributed to synergistic effects of multiple factors. Therefore, additional studies are needed for a comprehensive elucidation of the synergistic effects of multiple factors in the fish-killing activities of flagellate cells. For instance, the biochemical and cellular structural characteristics of *Chattonella* and the physiological vulnerability of gill tissue of susceptible fish species to the flagellates should be examined. Although studies have shown that *Chattonella* extracts possess several bioactive compounds with hemolytic [3,333] and antioxidant [334,335,336,337] activities, their biological significance is still an open question. Further efforts can help elucidate the exact roles of ROS and other bioactive molecules and the detailed processes leading to eventual fish death. Overall, current findings on *Chattonella* are summarized in a schematic diagram (Figure 1).

## Figures and Tables

**Table 1:** Harmful effect of HAB-forming species and their toxins (directly citing reviews of Landsberg [3]).

Species	Main Toxic Factors	Event	References
*Karenia mikimotoi*	Hemolysin; Reactive oxygen species (ROS)	Ichthyotoxic; Toxic to invertebrates; Toxic to zooplankton; Antialgal	[48,49,50,51,52,53,54,55,56]
*Cochlodinium polykrikoides*	Hemolysin; ROS; Sulfated polysaccharides; Noxiustoxin	Antiviral; Ichthyotoxic; Molluscicidal	[57,58,59,60,61,62]
*Eutreptiella gymnastica*	ROS	-	[62]
*Alexandrium tamarense*	Saxitoxin; Neosaxitoxin; Gonyautoxin; *N*-sulfocarbomoyl toxins; Tetrodotoxin; Hemolysin; ROS	Neurotoxic; Paralytic shellfish poisoning; Toxic to marine organisms; Toxic to zooplankton; Cytotoxic	[63,64,65,66,67,68,69,70,71,72,73,74,75,76,77,78,79,80,81,82,83]
*Heterosigma akashiwo (=Heterosigma carterae)*	Brevetoxin-like toxin; ROS	Ichthyotoxic; Neurotoxic; Toxic to zooplankton	[84,85,86,87,88,89]
*Chattonella marina*	Brevetoxin-like toxin ROS; Hemolysin; Hemagglutinin	Ichthyotoxic; Neurotoxic	[9,88,90,91,92,93,94]
*Chattonella ovata*	ROS	Ichthyotoxic	[95]
*Chattonella antiqua*	Brevetoxin-like toxin; ROS	Ichthyotoxic; Neurotoxic	[96,97,98,99,100]
*Chattonella subsalsa*	ROS; Hemolysin	Ichthyotoxic; Toxic to zooplankton	[101]
*Platymonas subcordiformis*	ROS	-	[102]
*Skeletonema costatum*	ROS	Ichthyotoxic; Toxic to zooplankton; Antibacterial	[103,104,105,106]
*Olisthodiscus luteus*	ROS	Ichthyotoxic; Antimycotic; Toxic to phytoplankton	[88,98,107,108]
*Fibrocapsa japonica*	Brevetoxin-like toxin; ROS; Fibrocapsin	Ichthyotoxic; Toxic to marine mammals; Neurotoxic; Toxic to phytoplankton	[88,107,109]
*Heterocapsa circularisquama*	ROS; Hemolysin	Toxic to mollusks; Antialgal; Antiprotozoal; Toxic to zooplankton	[110,111,112,113,114,115]
*Akashiwo sanguineum* *(=Gymnodinium sanguineum)*	ROS	Ichthyotoxic; Toxic to mollusks; Antimycotic; Toxic to mice	[55,98,116,117]
*Karlodinium veneficum*	ROS	Ichthyotoxic; Toxic to zooplankton	[55,118,119]
*Alexandrium catenella*	Saxitoxin; Neosaxitoxin; Gonyautoxin; *N*-sulfocarbomoyl toxins; Hemolysin; ROS	Neurotoxic; Paralytic shellfish poisoning; Toxic to marine organisms	[73,82,120,121,122,123,124,125,126]
*Prorocentrum minimum*	Venerupin; Prorocentrin; ß-diketone; ROS	Venerupin shellfish poisoning; Toxic to marine organism; Neurotoxic	[127,128,129,130,131,132,133,134,135,136,137,138,139]
*Prymnesium parvum*	Prymnesin 1 and 2; Hemolysin	Ichthyotoxic; Toxic to invertebrates; Toxic to tadpoles; Toxic to zooplankton; Cytotoxic	[140,141,142,143,144,145]
*Thalassiosira weissflogii*	ROS	Toxic to zooplankton	[105]
*Thalassiosira pseudonana*	Apo-fucoxanthinoid pigments; ROS	Toxic to zooplankton	[146,147]
*Coscinodiscus* sp.	ROS	-	[148]
*Pleurochrysis carterae*	ROS	Toxic to zooplankton	[149]
*Symbiodinium* spp.	ROS	-	[150]
*Trichodesmium erythraeum*	ROS	Antibacterial; Toxic to marine organisms; Hepatotoxic; Neurotoxic; Ciguatoxin-like	[151,152,153,154]
*Prorocentrum micans*	ROS	Shellfish poisoning; Toxic to marine organisms; Antialgal	[62,155,156,157,158]

**Table 2:** List of assay methods used for the detection of reactive oxygen species (ROS) in microalgae.

ROS	Studied Algal Species	Methods	References
Superoxide (O_2_^−^)	*Chattonella marina* *Chattonella antiqua* *Karenia mikimotoi* *Cochlodinium polykrikoides* *Chattonella ovata* *Olisthodiscus luteus*	^1^ MCLA-mediated chemiluminescence assay	[62,94,159,160,166,181,190,191,192]
	*Chattonella marina* *Chattonella antiqua* *Karenia mikimotoi* *Cochlodinium polykrikoides* *Chattonella ovata*	^2^ L012-mediated chemiluminescence assay	[164,193,194,195]
	*Cochlodinium polykrikoides* *Heterosigma carterae* *Thalassiosira weissflogii* *Thalassiosira pseudonana*	Cytochrome c-mediated spectrophotometric assay	[62,85,196]
	*Chattonella marina* *Nostoc spongiaeforme*	^3^ DMPO-mediated spin trapping method using an ESR	[159,197,198]
	*Trichodesmium erythraeum*	^4^ Red-CLA-mediated chemiluminescence	[199]
Hydroxyl radical (OH^−^)	*Chattonella marina*	Phenol red assay	[161]
Hydrogen peroxide (H_2_O_2_)	*Chattonella marina* *Cochlodinium polykrikoides*	^3^ DMPO-mediated spin trapping method using an ESR	[159,198]
	*Karenia mikimotoi* *Cochlodinium polykrikoides* *Chattonella ovata*	^5^ PHPA-mediated fluorescence spectrophotometric assay	[178,181,200]
	*Cochlodinium polykrikoides*	Scopoletin–peroxidase method	[62,201]
Nitric oxide	*Chattonella marina Cochlodinium polykrikoides*	Phenol red assay	[178,202]
	*Chattonella marina*	Luminol–H_2_O_2_-mediated luminescence assay	[185,203]
	*Chattonella marina* *Heterosigma akashiwo* *Chatonella ovata* *Cochlodinium polykrikoides* *Alexandrium taylori* *Alexandrium tamarense* *Nannochloropsis oculata*	^6^ DAF-FM DA-mediated fluorometric assay	[204]
Nitric oxide	*Platymonas subcordiformis* *Skeletonema costatum* *Gymnodinium* sp.	Nitric oxide detection microsensor	[102]

^1^ MCLA, methyl cypridina luciferin analog; ^2^ L012, 8-amino-5-chloro-7-phenylpyrido [3,4-d]pyridazine-1,4-(2H,3H)-dione; ^3^ DMPO, 5,5-dimethyl-1-pyrroline N-oxide; ^4^ red CLA, [2-[4-[4-[3,7-dihydro-2-methyl-3-oxoimidazo[1,2-*a*]pyrazin-6-yl]phenoxy]butyramido]ethylamino]sulforhodamine 101; ^5^ PHPA, *p*-hydroxyphenyl acetic acid; ^6^ DAF-FM DA, 4-Amino-5-methylamino-2′,7′-difluorofluorescein diacetate.

**Table 3:** Reactive oxygen species (ROS)-producing microalgae and the estimated production mechanisms.

Algal Species	ROS	Estimated Production Mechanisms	References
*Karenia mikimotoi*	Superoxide Hydrogen peroxide	-	[181,182]
*Cochlodinium polykrikoides*	Superoxide Hydrogen peroxide Hydroxyl radical	• Auto-oxidation of an electron acceptor in photosystem I (superoxide) • SOD catalyzed disproportionation of superoxide (hydrogen peroxide)	[159,178,198,217]
*Eutreptiella gymnastica*	Hydrogen peroxide	• SOD catalyzed disproportionation of superoxide (hydrogen peroxide)	[62,184]
*Prorocentrum micans*	Hydrogen peroxide	• SOD catalyzed disproportionation of superoxide (hydrogen peroxide)	[62]
*Akashiwo sanguineum* *(=Gymnodinium sanguineum)*	Hydrogen peroxide	• SOD catalyzed disproportionation of superoxide (hydrogen peroxide)	[62]
*Alexandrium tamarense*	Hydrogen peroxide	• SOD catalyzed disproportionation of superoxide (hydrogen peroxide)	[62]
*Heterosigma akashiwo* *(=Heterosigma carterae)*	Superoxide Hydrogen peroxide Hydroxyl radical Nitric oxide	• Glycocalyx-mediated ROS generation • SOD catalyzed disproportionation of superoxide (hydrogen peroxide)	[62,85,162,218]
*Chattonella marina*	Superoxide Hydrogen peroxide Nitric oxide	• NAD(P)H oxidase located in cell surface-bounded glycocalyx (superoxide) • SOD catalyzed disproportionation of superoxide (hydrogen peroxide) • Nitric oxide synthase-like enzyme-mediated mechanism (nitric oxide)	[94,162,185,203]
*Chattonella ovata*	Superoxide Hydrogen peroxide Nitric oxide	• NAD(P)H oxidase located in cell surface-bounded glycocalyx (superoxide) • SOD catalyzed disproportionation of superoxide (hydrogen peroxide)	[162]
*Chattonella antiqua*	Superoxide Hydrogen peroxide	• NAD(P)H oxidase located in cell surface-bounded glycocalyx (superoxide) • Photosynthetic electron transport (superoxide) • SOD catalyzed disproportionation of superoxide (hydrogen peroxide)	[193,219]
*Chattonella subsalsa*	Superoxide	• Photosynthetic electron transport (superoxide)	[217]
*Platymonas subcordiformis*	Superoxide	-	[102]
*Skeletonema costatum*	Superoxide	-	[102]
*Olisthodiscus luteus*	Superoxide Hydrogen peroxide	• Cell surface redox enzyme-mediated mechanism (superoxide) • SOD catalyzed disproportionation of superoxide (hydrogen peroxide)	[108]
*Fibrocapsa japonica*	Superoxide Hydrogen peroxide	-	[174]
*Heterocapsa circularisquama* *Karlodinium veneficum*	Hydrogen peroxide Superoxide	-	[182,184]
		-	
*Alexandrium catenella*	Superoxide	-	[184]
*Prorocentrum minimum*	Hydrogen peroxide	-	[184]
*Prymnesium parvum*	Superoxide	-	[184]
*Thalassiosira weissflogii*	Superoxide Hydrogen peroxide	• NAD(P)H oxidase-related mechanism	[196,220]
*Thalassiosira pseudonana*	Superoxide Hydrogen peroxide	• NAD(P)H oxidase-related mechanism	[196]
*Thalassiosira oceanica*	Superoxide Hydrogen peroxide	• NAD(P)H oxidase-related mechanism	[221]
*Coscinodiscus* sp.	Superoxide	-	[184]
*Pleurochrysis carterae*	Hydrogen peroxide	-	[222]
*Symbiodinium* spp.	Superoxide	-	[150,223]
*Trichodesmium erythraeum*	Superoxide	-	[199]

**Table 4:** The most harmful and notable HAB-forming species highlighted in this review.

Species	Main Toxic Factors Detected	Susceptible Organisms	Topics
*Chattonella* *marina/antiqua* (Raphidophyte)	1 ROS (superoxide, hydrogen peroxide, and hydroxyl radical) 2 Nitric oxide (NO)	Fish	1 NADPH oxidase is proposed as a mechanism of ROS production, which might be located on glycocalyx, a cell surface structure [94,162,193,219]. 2 The highest ROS generation rate among the species tested so far [184].
*Cochlodinium polykrikoides* (Dinoflagellate)	1 Hemolysin 2 ROS (superoxide and hydrogen peroxide)	Fish Shellfish	1 Secretion of huge amount of highly viscous mucus-like substances [61,276].
*Karenia mikimotoi* (Dinoflagellate)	1 Hemolysin 2 ROS (superoxide and hydrogen peroxide)	Fish Shellfish	1 Extremely toxic to both fish and shellfish, and HABs due to this dinoflagellate are often associated with mass mortality of both fish and shellfish [279,299].

## Data Availability

The datasets used and analyzed during the current study are available from the corresponding author on reasonable request..
